# Severe Hypercapnia and Hemodynamic Instability During Carbon Dioxide Insufflation During Robot-Assisted Thoracic Surgery

**DOI:** 10.7759/cureus.117142

**Published:** 2026-09-29

**Authors:** Mototsugu Watanabe, Noriaki Shiraha, Yoshinobu Shikatani, Kenji Uehara

**Affiliations:** 1 Thoracic Surgery, National Hospital Organization Iwakuni Clinical Center, Iwakuni, JPN; 2 Anesthesia, National Hospital Organization Iwakuni Clinical Center, Iwakuni, JPN

**Keywords:** atrial fibrillation, co₂ insufflation, hemodynamic instability, hypercapnia, robot-assisted thoracic surgery

## Abstract

The carbon dioxide (CO_2_) insufflation system enhances robot-assisted thoracoscopic surgery (RATS) by maintaining a stable capnothorax and facilitating smoke evacuation. However, CO_2_ insufflation can cause hypercapnia and hemodynamic instability, requiring vigilant monitoring. We report a case examining the severity and time course of CO_2_-induced hypercapnia during capnothorax-assisted robot-assisted thoracoscopic surgery (RATS). A 68-year-old obese man underwent robot-assisted lung resection with capnothorax, resulting in elevated CO_2_ levels that caused atrial fibrillation and hypotension. Blood gas analysis showed severe acidosis and hypercapnia. Following discontinuation of the capnothorax, hypercapnia improved within 37 minutes. The surgery was completed successfully without capnothorax, and the patient was discharged on postoperative day 6 without complications. While CO_2_ insufflation supports minimally invasive surgery, this case documents an extreme hypercapnic event with an arterial partial pressure of carbon dioxide (PaCO_2_) of 110 mmHg with rapid cardiovascular compromise in a high-risk patient using the capnothorax system, underscoring the need for vigilant monitoring, even with advanced insufflation technology.

## Introduction

The use of carbon dioxide (CO_2_) insufflation, or capnothorax, has become increasingly common in robot-assisted thoracic surgery (RATS) to optimize the operative field. Problems associated with CO_2_ insufflation systems include impaired venous return and cardiac filling due to CO_2_ accumulation, as well as increased intrathoracic pressure. The AirSeal® insufflation system (CONMED Corporation, Utica, NY, USA) represents a significant advancement in this technology by providing continuous smoke evacuation and stable pressure control. These features enhance visualization and ensure a consistent working space, contributing to procedural efficiency [1,2]. However, the physiological effects of CO_2_ insufflation, particularly during one-lung ventilation (OLV), require careful monitoring. This case report examines the severity of CO_2_-induced hypercapnia during AirSeal-assisted RATS in high-risk patients, and the time course of its resolution after cessation of insufflation. We describe a life-threatening hypercapnic episode with an arterial partial pressure of carbon dioxide (PaCO₂) of 110 mmHg that improved within 37 minutes, highlighting the critical importance of vigilant physiological monitoring and patient-specific risk assessment.

## Case presentation

We present the case of a 68-year-old man who was referred for evaluation of a lung nodule in the right lower lobe during routine check-ups for kidney stones. His medical history included several conditions: hypertension, diabetes mellitus, hypercholesterolemia, hyperuricemia, ossification of the posterior longitudinal ligament, and cerebrospinal fluid leakage. Prior to admission, his activities of daily living were fully independent, and his Hugh-Jones classification was class I. With a BMI of 30.2 kg/m^2^, indicating obesity, and a 48-year smoking history of 20 cigarettes per day, pulmonary function tests showed a forced vital capacity (FVC) of 3.52 L, vital capacity (VC) of 3.79 L, and %VC, an actual VC/VC expected from age and height, of 106.9%. The forced expiratory volume in one second (FEV_1.0_) was 2.32 L, and the FEV_1.0_/FVC ratio was 65.76%. These findings indicated obstructive ventilatory impairment and an increased risk of postoperative pulmonary complications. These factors necessitated thorough preoperative evaluation and careful perioperative management during the planned robot-assisted lung surgery.

Computed tomography (CT) and fluorodeoxyglucose positron emission tomography (FDG-PET) findings indicated a preoperative diagnosis of cT1cN0M0, stage IA3, according to the 9th edition of the lung cancer staging system (Figure 1). These findings supported the decision to proceed with surgery.

**Figure 1:**
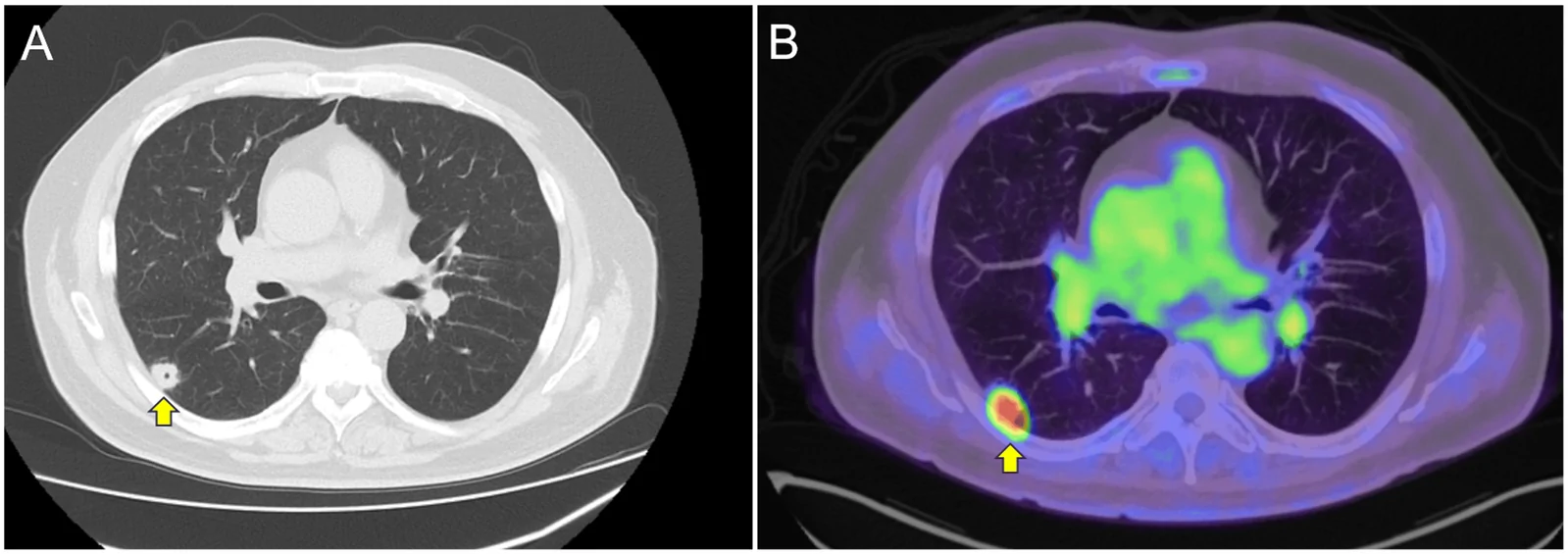
Chest computed tomography (CT) and fluorodeoxyglucose positron emission tomography (FDG-PET) findings. (A) CT revealed a 2.2 cm mass (yellow arrow) in the right lower lobe. (B) FDG-PET demonstrated substantial accumulation of FDG in the tumor (yellow arrow). No distant metastasis was detected.

The surgical procedure involved a robot-assisted partial resection of the right lower lobe, and if lung cancer was diagnosed, lobectomy with systematic lymph node dissection was planned. After general anesthesia under pressure control of 10 cmH₂O, tidal volume was maintained at approximately 300-500 mL, positive end-expiratory pressure of 4 cmH₂O at a respiratory rate of 12 breaths per minute was induced, and a 35 Fr. double-lumen endotracheal tube was placed for OLV; the initial arterial blood gas (ABG) analysis showed a pH of 7.46 and PaCO_2_ of 28.3 mmHg. The AirSeal system was initiated with an insufflation pressure of 8 mmHg and a CO_2_ flow rate of 3 L/min. Initially, the CO_2_ capnothorax was well tolerated, but CO_2_ levels gradually increased during the operation. The anesthesiologist was continuously positioned at the head of the bed, strictly monitoring tube placement and the patient's vital signs. The ventilator settings were adjusted by increasing the respiratory rate; however, the end-tidal CO_2_ level continued to rise. This led to the sudden onset of atrial fibrillation and hypotension, raising concerns regarding the patient's stability. ABG analysis confirmed severe acidosis (pH 7.05) and hypercapnia (PaCO_2_ = 110.0 mmHg). At the same time, the patient developed atrial fibrillation with a rapid ventricular response (heart rate > 150 bpm) and significant hypotension (blood pressure 70/50 mmHg). The surgical team promptly halted the procedure and deactivated the AirSeal system. The hypotensive episode occurred immediately after the onset of atrial fibrillation and persisted even after the cessation of CO_2_ insufflation. Therefore, the decrease in blood pressure was unlikely to be attributable to increased intrathoracic pressure. Hypercapnia resolved after 37 min. After discontinuing CO_2_ insufflation, oxygenation rapidly normalized, and end-tidal carbon dioxide (EtCO_2_) levels decreased. This clinical course strongly suggests that CO_2_ administration was a primary contributing factor to the event. A repeat ABG after 15 minutes showed a pH of 7.05 and a PaCO_2_ of 97.0 mmHg, and after 37 minutes, a pH of 7.15 and a PaCO_2_ of 74.0 mmHg, confirming the improvement of severe hypercapnia and acidosis (Figure 2).

**Figure 2:**
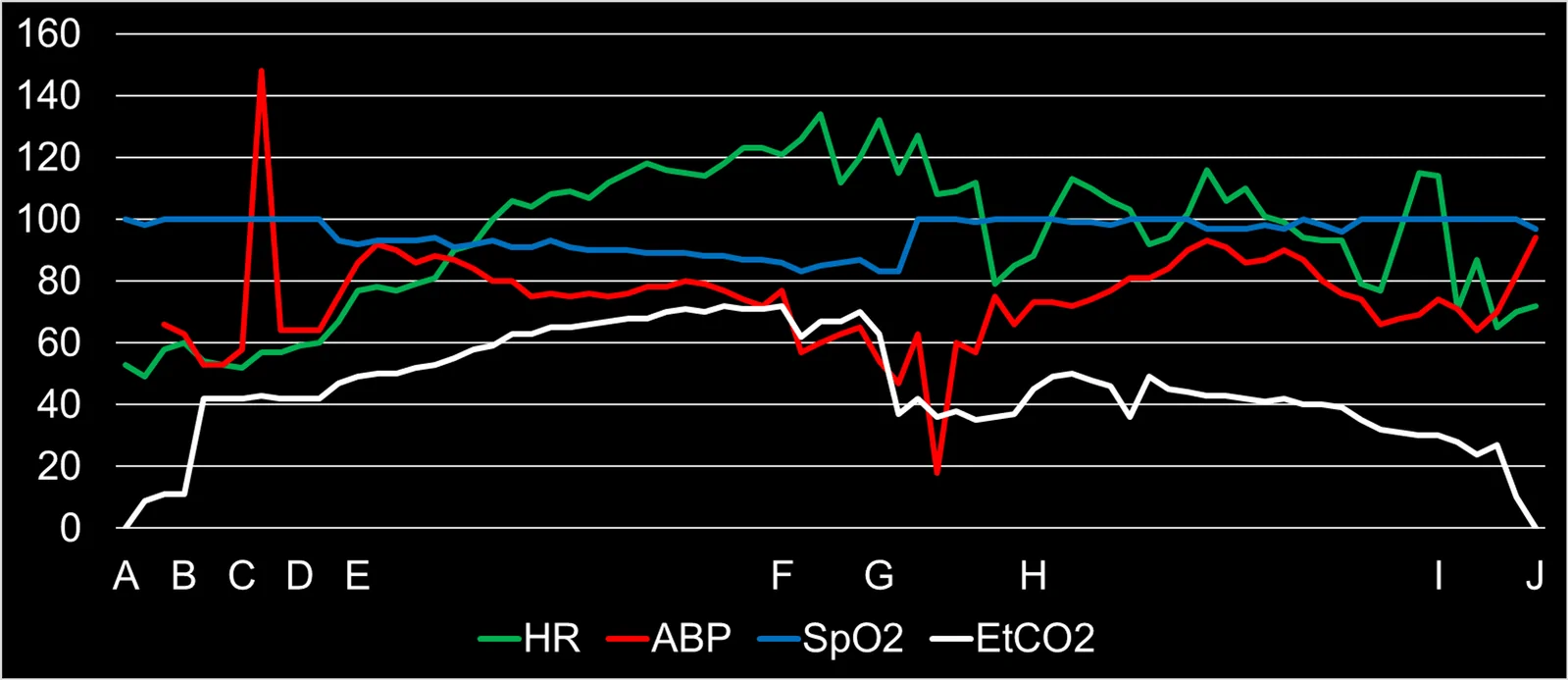
Intraoperative anesthesia record. (A) Anesthesia initiated; (B) left lateral position; (C) oxygen inhalation initiated; (D) one-lung ventilation initiated; (E) CO_2_ insufflation initiated; (F) atrial fibrillation occurred; (G) CO_2_ insufflation ceased and surgery interrupted; (H) surgery resumed without CO_2_ insufflation; (I) surgery completed; (J) anesthesia ended. HR: heart rate, ABP: arterial blood pressure, SpO_2_: peripheral oxygen saturation, EtCO_2_: end-tidal carbon dioxide.

The surgery was completed without resuming CO_2_ insufflation, thereby ensuring patient safety. The chest tube was removed on postoperative day 3, and the patient was discharged on day 6 without long-term complications, indicating good recovery. At the one-year postoperative follow-up, the patient remained in good clinical condition, with no evidence of long-term sequelae related to the intraoperative hypercapnic event.

## Discussion

The induction of severe hypercapnia and subsequent hemodynamic instability is a well-documented risk associated with CO_2_ insufflation during thoracic surgery, irrespective of the delivery system employed. During OLV, the diminished surface area available for gas exchange, coupled with the potential for intrapulmonary shunting, restricts the patient's capacity to expel CO_2_. Additionally, the pleural surface serves as an extensive area for CO_2_ absorption. When CO_2_ is introduced into the thoracic cavity to establish capnothorax, systemic absorption can rapidly exceed the compensatory capability of the respiratory system, resulting in respiratory acidosis and cardiovascular disturbances, such as arrhythmias and hypotension [3-5].

The risk is significantly heightened by patient-specific factors. In this case, the patient's obesity and long history of smoking were significant risk factors. Obesity reduces functional residual capacity and increases airway resistance, whereas chronic smoking negatively impacts baseline pulmonary function. The interaction of these factors with the physiological demands of OLV substantially impairs CO_2_ clearance [6].

In the context of CO_2_-related risks, the AirSeal system offers specific technical advantages but requires precautionary measures to ensure safety. To our knowledge, this is one of the first detailed reports of life-threatening hypercapnia specifically associated with AirSeal use in RATS, highlighting that advanced insufflation technology does not eliminate physiological risk. Unlike traditional insufflators, the AirSeal system's valveless design and high-flow capacity maintain stable intrathoracic pressure while allowing continuous smoke evacuation [1,2]. These features help maintain a clear operative field and may reduce operative time during complex robotic procedures. However, the system's ability to maintain pressure, even with minor suctioning or leaks, may affect total CO_2_ delivery, although we did not measure this parameter. Comparative studies directly measuring CO_2_ delivery across insufflation systems are required to test this hypothesis. This stable pressure could enable continuous transpleural CO_2_ absorption, potentially leading to rapid hypercapnia if not carefully monitored. However, this case cannot distinguish AirSeal-specific effects from the patient's obesity, smoking history, and obstructive lung disease, all of which independently impair CO_2_ clearance during surgery.

The cardiovascular manifestations in this patient, notably atrial fibrillation and hypotension, highlight the severity of systemic acidosis in this condition. Sympathetic activation due to hypercapnia, followed by myocardial depression, can lead to life-threatening arrhythmias and hemodynamic collapse [7,8]. In the present case, hemodynamic instability occurred immediately following the onset of atrial fibrillation and persisted even after the cessation of CO_2_ insufflation. Therefore, the hypotensive episode was unlikely to be attributable to elevated intrathoracic pressure; rather, it was thought to be secondary to hypercapnia. Although arrhythmia was present in this case, a sudden hypotension may occur even in the absence of preceding electrocardiographic changes [9]. In line with previous reports, the present case exhibited a sudden electrocardiographic change, highlighting the necessity of a multidisciplinary approach involving anesthesiologists, medical technologists, and other medical staff. Anesthesiologists must maintain heightened vigilance through continuous capnography and frequent ABG analysis, and surgeons should be prepared to reduce or discontinue insufflation immediately if physiological parameters deteriorate.

## Conclusions

Ultimately, although advanced insufflation systems such as AirSeal enhance the technical precision of RATS, they do not eliminate the inherent risks associated with CO_2_ insufflation. The potential risks associated with CO_2_ insufflation during RATS include hypercapnia and respiratory acidosis, hemodynamic instability, gas embolism, and barotrauma. This single-case report cannot establish causality or generalize the risk across patient populations; therefore, larger comparative studies are needed to quantify AirSeal-specific versus patient-specific contributions to hypercapnia. A comprehensive preoperative risk assessment for patients with obesity or pulmonary comorbidities, along with vigilant intraoperative monitoring, remains crucial to ensure patient safety during capnothorax.
